# Finding and treating both tuberculosis disease and latent infection during population-wide active case finding for tuberculosis elimination

**DOI:** 10.3389/fmed.2023.1275140

**Published:** 2023-10-16

**Authors:** Mikaela Coleman, Thu-Anh Nguyen, Boi Khanh Luu, Jeremy Hill, Romain Ragonnet, James M. Trauer, Greg J. Fox, Guy B. Marks, Ben J. Marais

**Affiliations:** ^1^WHO Collaborating Centre for Tuberculosis and the Sydney Infectious Diseases Institute (Sydney ID), The University of Sydney, Sydney, NSW, Australia; ^2^Centenary Institute, The University of Sydney, Sydney, NSW, Australia; ^3^Woolcock Institute of Medical Research, Hanoi, Vietnam; ^4^Faculty of Medicine and Health, The University of Sydney, Sydney, NSW, Australia; ^5^School of Public Health and Preventive Medicine, Monash University, Melbourne, VIC, Australia; ^6^Woolcock Institute of Medical Research, Sydney, NSW, Australia; ^7^Department of Medicine and Health, University of New South Wales, Sydney, NSW, Australia

**Keywords:** *Mycobacterium tuberculosis*, TB, latent infection, TPT, LTBI, active case finding, systematic screening, prevention

## Abstract

In recognition of the high rates of undetected tuberculosis in the community, the World Health Organization (WHO) encourages targeted active case finding (ACF) among “high-risk” populations. While this strategy has led to increased case detection in these populations, the epidemic impact of these interventions has not been demonstrated. Historical data suggest that population-wide (untargeted) ACF can interrupt transmission in high-incidence settings, but implementation remains lacking, despite recent advances in screening tools. The reservoir of latent infection—affecting up to a quarter of the global population –complicates elimination efforts by acting as a pool from which future tuberculosis cases may emerge, even after all active cases have been treated. A holistic case finding strategy that addresses both active disease and latent infection is likely to be the optimal approach for rapidly achieving sustainable progress toward TB elimination in a durable way, but safety and cost effectiveness have not been demonstrated. Sensitive, symptom-agnostic community screening, combined with effective tuberculosis treatment and prevention, should eliminate all infectious cases in the community, whilst identifying and treating people with latent infection will also eliminate tomorrow’s tuberculosis cases. If real strides toward global tuberculosis elimination are to be made, bold strategies are required using the best available tools and a long horizon for cost-benefit assessment.

## Introduction

After the Second World War the tuberculosis control agenda in most high-income countries was strongly focused on active case finding (ACF). This focus led to a twenty-fold decline in tuberculosis incidence and near elimination in many areas that formerly had a high tuberculosis incidence (1–5). Once tuberculosis was considered “conquered”—with low disease rates in high-income countries and effective treatment widely available—governments shifted their investment and the health policy focus to other priorities. However, tuberculosis has continued to plague low-income countries where it has never been “conquered” and where community transmission often remains uncontrolled. The DOTS (directly observed therapy short course) strategy of rolling out passive case finding linked to effective treatment of sputum smear-positive tuberculosis has contributed to substantial reductions in tuberculosis-related mortality over the past 20 years. However, this strategy had limited epidemic impact (6). Nevertheless, the implementation of better tuberculosis data collection and reporting systems is an important legacy of the DOTS strategy. Today, progress envisioned by the 2014 World Health Organization (WHO) End TB strategy has been hampered by major setbacks in global tuberculosis control resulting from the coronavirus 2019 (COVID-19) pandemic. Therefore, it is now imperative to reevaluate what would be required to truly “move the dial” toward reducing tuberculosis transmission in high incidence settings and achieving global tuberculosis elimination.

It is increasingly appreciated that asymptomatic or “subclinical” tuberculosis (where people are infectious but do not feel ill enough to present to the healthcare system) contributes to a large proportion of community transmission (7). This implies that traditional passive case finding, where programs wait on a symptomatic patient to present to the healthcare system for diagnosis and treatment, is simply insufficient to have a community-wide transmission impact (8). Furthermore, it is estimated that more than a third of active tuberculosis cases every year remain undetected and so are absent from surveillance data (9). In addition, latent tuberculosis infection is now considered a dynamic state, at least in some people, with waxing and waning bacilli numbers and highly variable risk of reactivation (10–13). Under an elimination paradigm, it is prudent to regard latent tuberculosis infection as a “risk state” that carries the potential both to lead to individual harm and to perpetuate community transmission (14, 15). This multifaceted challenge of asymptomatic tuberculosis transmission combined with persistent low rates of disease detection and the risk of reactivation from a latent reservoir constitutes a complex epidemiological reality. This complexity may explain why simple strategies for global tuberculosis elimination remain elusive. Holistic, combined approaches targeting both active and latent disease in a symptom-agnostic way may provide an opportunity to recapture the successes of the past in the modern era.

### Population-wide active case finding

Interventions that increase timely access to healthcare for tuberculosis patients, together with good oversight and quality control systems, have typically been emphasized to ensure that people with tuberculosis are diagnosed early and treated appropriately. However, the 2022 WHO Global Report indicates that—despite this long-held emphasis—the decline in tuberculosis incidence is far below End TB targets for most high burden countries (9). In these settings, people with “classic” tuberculosis symptoms do not always seek healthcare and even if they do so, poor access to healthcare, insensitive tuberculosis testing, social stigma and financial insecurities often impede timely and effective treatment (16). The WHO has estimated that in 2021, only 60% of tuberculosis patients globally were identified and notified to the healthcare system (9). Hence, active case finding has been strongly recommended to find and treat unreached tuberculosis patients (17). The primary focus has been on specific high-risk populations, including household contacts of index patients, people living with HIV, workers exposed to silica and incarcerated people (18–24). Although active case finding (ACF) among these “risk groups” is effective in finding new cases, such activities are insufficient to effectively interrupt tuberculosis transmission or reduce disease prevalence on a population scale (25). This is because a significant proportion of infectious tuberculosis cases, particularly in high incidence settings, occur in patients with none of these identifiable risk factors (7, 26, 27). In addition, such targeted case finding fails to address the large pool of people with latent tuberculosis infection, representing a reservoir which can continue to seed new active tuberculosis cases and perpetuate transmission for decades to come (28, 29).

Settings in which transmission had historically been high and where mass radiography screening was subsequently conducted (such as North America, Australia, and the United Kingdom) observed dramatic reductions in tuberculosis incidence and mortality following the introduction of these programs (1–5). Recently, a community-based cluster randomized controlled trial of active case finding for tuberculosis (the ACT3 study) was conducted in Ca Mau, a rural province in the Mekong Delta region of Vietnam (30). Starting in 2014, ACT3 aimed to evaluate the effectiveness of repeated community-wide screening over 3 years, as compared with standard passive case detection alone, for reducing the prevalence of tuberculosis. By the conclusion of the study, adult tuberculosis prevalence was 44% lower and prevalence of tuberculosis infection (positive interferon gamma release assay) among children aged 6 to 14 years in the actively screened clusters was 50% lower than in the control clusters (30, 31). This trial suggests that if all people with tuberculosis disease are found and treated, then it is possible to drastically reduce community transmission (32). However, not all active case finding interventions have found a similarly dramatic effect and the high-uptake of community-wide tuberculosis screening required may be hard to replicate in all high-incidence settings (20, 33). However, multiple implementation studies are underway seeking to identify the most pragmatic strategies in a wider array of real-life settings, which are discussed further below.

### Treating latent tuberculosis infection

Latent tuberculosis infection (LTBI) is a state of persistent immune response to stimulation by *Mycobacterium tuberculosis* antigens, without evidence of clinically manifest disease (14). Individuals with LTBI have an increased lifetime risk of subsequent progression to active tuberculosis, although this risk is highly variable and concentrated in the period soon after infection or re-infection (10, 34). Globally, the prevalence of LTBI has been estimated to be ∼25% (95% uncertainty interval 20–26%), based upon historical cross-sectional surveys and tuberculosis prevalence estimates (35). However, substantial variation occurs between different populations on account of the differing risks of transmission. The tests used to diagnose LTBI [tuberculin skin test (TST) and interferon gamma release assays (IGRAs)] have significant limitations in their accuracy and ability to predict subsequent incident disease (36, 37). Diagnostic accuracy was poorer among studies from high-incidence settings (38) and no reference standard exists to assess accuracy for LTBI detection. In general, both tests are associated with a similar risk of future tuberculosis disease progression when positive, although even in the absence of tuberculosis preventive treatment (TPT) most people with a positive result never develop disease. The estimated positive predictive value of future TB disease for a positive TST is 2.75% and for a positive Quantiferon Gold is 2.46% (38).

Latent tuberculosis infection can be treated using 1 to 9 months of oral antibiotics (14), which reduce the incidence of tuberculosis by approximately two thirds compared to placebo. However, all available regimens may cause side effects and require careful clinical monitoring, which is a particular disincentive in people who are otherwise well. Consequently, the decision to treat individuals with LTBI requires careful evaluation of the risks of preventive treatment against the benefits to the affected individuals and their communities. This presents an ethical dilemma since the short-term individual benefit from reduced tuberculosis risk may be far less than the long-term community benefit of tuberculosis elimination, while the individual carries all the risk associated with preventive treatment. WHO currently recommends screening of identified high-risk populations, such as people living with HIV, recent household contacts and other groups, such as people initiating immunosuppressive treatment considered to be at high risk of tuberculosis disease progression (14). Whether additional groups can be identified who will benefit from LTBI-linked ACF in high-incidence settings remains under evaluation (10, 39). Three studies in the Asia-Pacific are currently underway to investigate this evidence gap including a randomized controlled trial and “real-life” implementation study, explored below. While evidence for its general implementation is not yet conclusive, combined systematic TB screening and LTBI detection provides an opportunity to strengthen the overall characterization of TB disease (especially early and asymptomatic disease) and transmission within communities.

### Current and future studies combining population-wide active case finding and prevention

A recent modeling study estimated the effects of population-wide ACF in the Marshall Islands (40). The analysis emphasized the crucial role of including LTBI screening and TPT as part of the intervention, in order to achieve significant and sustained reductions in tuberculosis burden. The findings also suggested that by implementing these strategies repeatedly, local tuberculosis pre-elimination could be achieved using existing tools alone. Additionally, the estimated number of severe adverse events associated with preventive treatment—a weekly dose of rifapentine and isoniazid for 12-weeks (3HP regimen)—was minimal compared to the significant reductions in tuberculosis deaths and disease episodes resulting from the interventions. The study highlighted another significant advantage of incorporating LTBI screening in ACF programs, as such approaches provide direct measures of LTBI prevalence in addition to those of active tuberculosis prevalence, which increases the richness of the dataset and allows measurement of transmission impact over time. The combination of these two indicators enables a more accurate characterization of the risk of future tuberculosis disease following exposure and infection, a factor that is essential for refining modeling projections and identifying the tuberculosis control approaches that will achieve the greatest reductions in tuberculosis burden.

The ACT5 trial is a randomized controlled trial currently assessing the effectiveness of universal testing and treatment for LTBI, together with ACF for tuberculosis disease, on the population prevalence of tuberculosis disease and ongoing community transmission in a high incidence setting in Vietnam. The overall goal of this trial is to acquire evidence that will underpin a transformation in the global approach to tuberculosis elimination in low and middle-income countries with a high burden of tuberculosis. Results of the trial will be available in 2026 (trial registration number ACTRN12622000115730).

Another example aiming to assess the impact of combining ACF and TPT at population level is the PEARL [Pathway to the Elimination of Antibiotic Resistant and Latent Tuberculosis (and also Leprosy) in the Pacific] study conducted in South Tarawa, Kiribati (39, 41). In this program, ACF is delivered using the best available combination of technologies (symptoms screening, mobile digital chest radiograph with computer-aided detection and sputum Xpert MTB/RIF Ultra^®^ on all who can expectorate) along with TPT (using short course regimens) for those who are TST positive. The impact of this ambitious public health intervention on case notifications will be compared to case notification rates before the intervention and trends in the rest of country where the intervention was not delivered. In addition, TB transmission impact will be assessed by comparing TST positivity in primary school aged children before and after the intervention. As with the Vietnam studies, a broader goal is to provide practical examples of how this active population-based approach can be effectively scaled in geographically diverse high-incidence locations, especially in remote Pacific settings. These and similar studies should provide insights into the value-add of combining TB prevention with community-wide active TB case-finding efforts. Given the complexity of such activities, these studies are essential to assess the feasibility of population-wide screening for infection and disease in resource-limited settings.

### Challenges of combining active tuberculosis case finding and prevention

Challenges facing the expansion of screening and treatment for LTBI in a community-wide fashion include the relatively high prevalence of remote past infection for which LTBI treatment will have reduced benefit, ethical concerns regarding the administration of a potentially toxic medication to someone for whom the individual benefit may be limited and the highly variable risk of future re-infection against which past LTBI treatment will offer no protection. Studies are ongoing to explore the feasibility, effectiveness and acceptability of large-scale screening and treatment for LTBI as a part of community-wide tuberculosis elimination efforts. Further considerations of the risks and benefits of combining ACT and TPT are highlighted in Table 1. Careful consideration must be given to contexts where dual active and latent tuberculosis screening and treatment is appropriate, but potential risks of over-diagnosis should be weighed against the higher risk of disease and death in high-incidence settings.

**TABLE 1:** Risks and benefits of combining tuberculosis ACF and TPT in high incidence settings.

Consideration	Risk	Benefit
Adverse reactions to TPT	Limited benefit of TPT to infected individuals who may have a low individual risk of developing disease and may experience adverse reactions to preventive treatment	Safer, shorter and better tolerated TPT regimens have led to decreased risk of adverse effects (55, 56). TPT coupled with ACF offers community-level benefits (as well as individual-level benefits for high progression risk groups) by minimizing the pool of latent infection that may reintroduce active disease into the population after detection and treatment of active cases by ACF (57).
Risk of reinfection after TPT	Limited value of TPT in settings with uncontrolled transmission due to high likelihood of reinfection and attendant risk of progression to disease (highest in the first 1–2 years) post reinfection.	When TPT is delivered in combination with repeated ACF rounds, community transmission is reduced (30), decreasing the risk of reinfection. ACF may also sensitize the community to signs and symptoms of active disease, strengthening early passive detection pathways and reducing the amount of time active cases may be transmitting in the community (58, 59).
High cost of “universal screening”	ACF is expensive, particularly if repeat activities are envisaged. Costs are compounded by the addition of TPT.	Tuberculosis ACF is highly cost-effective over a lifetime horizon, due to long-term diminution in tuberculosis mortality, amelioration of tuberculosis burden on healthcare systems, increased economic and social contributions of affected people and reduced catastrophic costs to families (50–52)
High complexity and limited capacity	High complexity and resource demands of combined interventions may make implementation unfeasible	Simpler and more field-ready diagnostic tools, shorter and safer LTBI regimens and context-specific algorithms for screening make ACF and TPT increasingly accessible for low-resource settings.
Distraction from routine program activities	ACF may shift the focus of TB control programs away from the identification, treatment and follow-up of the most ill patients (who presented passively), with worse patient outcomes	ACF activities typically lead to greater community awareness and may increase passive case detection (30). ACF also involves increased training and capacity building that ideally strengthens the existing control program (58, 59). Furthermore, the aim of these activities is to reduce the burden on routine program by driving disease rates toward elimination, which may ultimately lead to a diminution in need for services.
Low uptake amongst healthy people	Community-based screening that target the general population, regardless of symptoms or risk factors, may have substantially lower proportions of screening and treatment uptake if patient and/or healthcare providers perceive low tuberculosis risk or mistrust the screening test results.	Peer driven communication, thorough branding and marketing for screening programs, and widening screening areas can help to improve screening and treatment acceptance rates (60–63). The whole of community approach may also provide an opportunity for testing with reduced perceptions of community- and self-stigma (64).
Population-wide focus may diminish outreach to known risk groups	Offering community-wide TPT rather than targeting known risk groups may lead to oversight of the most affected groups	In high-burden settings, adolescents and young adults are some of the most populous groups and also at highest risk of disease and most likely to transmit infection (32, 48, 65–67). When those most at risk are also the largest population subgroup, population-wide ACF activities are highly appropriate.
Loss to follow up along the TPT cascade of care	Low TPT completion rates may limit the transmission impact of community-wide TPT in practice (68).	Risks of attrition from the TPT cascade of care may be minimized when individuals are actively tested and followed up for TPT as part of community-wide screening, especially as most “loss to follow up” occurs at the early stages of identification, referral for eligibility assessment and treatment commencement (68), all of which can be curtailed with ACF activities.
Over-diagnosis and over-treatment	ACF may lead to false positives and treatment of people who do not truly have tuberculosis infection or disease	Potential risks of over-diagnosis are likely to be significantly lower than the risk of tuberculosis mortality and morbidity in high-incidence settings is ACF if not implemented (8, 69).
Diagnosis of non-tuberculosis conditions	Tuberculosis ACF may lead to detection of other conditions that may impart an ethical duty to link patients to care in settings where health systems may be ill-equipped to provide this care	The likelihood of detecting unrelated conditions should not limit tuberculosis ACF activities given the high risks of ongoing tuberculosis transmission in the community, and the fact that many detected conditions will be routinely accommodated in local health systems with positive outcomes for participants. Intentional integration of tuberculosis ACF and TPT with multi-disease screening and healthcare provision was considered to increase uptake of tuberculosis screening, community acceptability and reduce tuberculosis-related stigma in settings where integrated ACF was employed (70–74), suggesting that detection of many conditions may benefit tuberculosis ACF and TPT uptake in some communities.
Emergence of drug resistant tuberculosis	Widespread use of antibiotics with potentially suboptimal completion rates may exacerbate the emergence of drug resistant tuberculosis strains	Most drug resistant tuberculosis is acquired by transmission rather than by *de novo* emergence, suggesting that reduced transmission should be the primary goal in the prevention of DR-TB (75–77). To eliminate the *de novo* emergence of drug resistant tuberculosis, drug sensitive tuberculosis must be eliminated (78).

ACF, active case finding; DR-TB, drug-resistant tuberculosis; LTBI, latent tuberculosis infection; TPT, tuberculosis preventive treatment.

### Progressing the tuberculosis elimination agenda

The current slow progress toward ending tuberculosis in high-incidence settings implies that more needs to be done to actively “move the dial” toward this goal (42). Several observations about the epidemiology of the global epidemic and the adequacy of existing healthcare provision are relevant:

1. Prevalence surveys have demonstrated that many people with bacteriologically confirmed pulmonary tuberculosis do not have overt symptoms and hence are unlikely to seek care or be diagnosed using a passive case finding approach (26, 43–45). They can only be found through active screening.
2. People who do have symptoms of tuberculosis (cough, fever or weight loss) are most likely to attend primary healthcare providers (46, 47), but these are common symptoms in persons without tuberculosis and primary health services in many low-middle income countries are fragmentary and poorly equipped to diagnose tuberculosis.
3. In settings with a high-incidence of TB, most people with tuberculosis are not in an identifiable high-risk group (32). In these settings everyone is at risk, all the time. Hence, targeted case finding or limited treatment of latent tuberculosis infection is unlikely to make a major contribution toward ending the epidemic (48).
4. There is evidence that re-infection with tuberculosis occurs commonly in high-incidence settings and that previous tuberculosis infection or disease does not protect against subsequent reinfection. In fact, people who have had tuberculosis in the past are more likely to become diseased again if reinfected (49). Hence, the benefit of treatment for tuberculosis infection for a person who lives in a high tuberculosis incidence setting is likely to be limited and transient unless simultaneous efforts are made to eliminate community transmission at the same time in order to substantially reduce that person’s risk of re-exposure and, hence, reinfection.

It follows that, to reduce the incidence of new cases of tuberculosis in high incidence settings and make substantial progress toward ending tuberculosis, it is necessary to drastically reduce the number of persons with untreated (and usually undiagnosed) tuberculosis who sustain transmission within communities (7). Since these people will not present for care and are not identifiable by specific risk factors in high tuberculosis incidence settings, it is necessary to screen (test) everyone for tuberculosis using a symptom-agnostic test (either radiology or a molecular test on sputum) as the front-line screening test. It is also essential that those who are diagnosed with tuberculosis as a result of this screening are linked to effective care, given appropriate treatment and are adequately supported to complete their treatment.

The role of mass treatment of tuberculosis infection in this approach remains to be fully elucidated. In theory, it should expedite the ending of endemic transmission, when linked to screening for active disease, by averting development of tuberculosis in those who were recently infected. Several studies to clarify the role of mass treatment of tuberculosis infection in tuberculosis elimination are currently underway. Community-wide screening for tuberculosis disease, with or without mass treatment of tuberculosis infection, is a major logistical and financial challenge for tuberculosis programs, Ministries of Health and funders. Importantly, these activities may be cost saving by reducing the load of passive cases on the health system when considered over a longer time horizon. More work is required to establish the best way to implement these programs at scale. As tuberculosis is a slowly evolving condition, some people who were recently infected at the time of screening may only develop tuberculosis over the next 1 to 2 years and longer. There is also a conversion window of 6–8 weeks for TST and IGRA test to register recent TB infection and sensitivity of these tests is suboptimal. In addition, even comprehensive population-wide screening is likely to miss a sizable part of the population, which may include those at highest risk of LTBI and tuberculosis disease. This means that screening interventions may have to be repeated, until the disease prevalence has fallen to a low enough level so that ongoing endemic transmission can be prevented through more targeted means.

## Conclusion

Effective tuberculosis elimination strategies have challenges and are daunting to implement, but with 23.8 million avoidable tuberculosis deaths predicted to occur globally by 2035 if current downward trends in tuberculosis incidence are not accelerated (50), the cost of inaction is high. Bold strategies are required to “move the dial” towards elimination, which would not only save lives lost to tuberculosis, but will also reduce pressure on fragile healthcare systems. Despite the need for considerable up-front investment, strategies that effectively reduce community transmission and make real progress toward tuberculosis elimination will ultimately save money, if a sufficient time horizon is adopted for the analysis (51–54). Recent transmission insights, technological advances, funding commitments and increased international political will, create new opportunities to bring tuberculosis elimination within our grasp. It is therefore prudent to reflect whether tried and tested active case finding strategies combined with modern advances can be leveraged to replicate and even accelerate historical achievements in tuberculosis control.

## Author contributions

MC: Conceptualization, Writing – original draft, Writing – review and editing. T-AN: Conceptualization, Writing – original draft, Writing – review and editing. BL: Writing – original draft. JH: Writing – original draft. RR: Writing – original draft. JT: Writing – review and editing. GF: Writing – original draft, Writing – review and editing. GM: Writing – original draft. BM: Conceptualization, Writing – original draft, Writing – review and editing.
